# MUC1-C integrates activation of the IFN-γ pathway with suppression of the tumor immune microenvironment in triple-negative breast cancer

**DOI:** 10.1136/jitc-2020-002115

**Published:** 2021-01-24

**Authors:** Nami Yamashita, Mark Long, Atsushi Fushimi, Masaaki Yamamoto, Tsuyoshi Hata, Masayuki Hagiwara, Atrayee Bhattacharya, Qiang Hu, Kwok-Kin Wong, Song Liu, Donald Kufe

**Affiliations:** 1 Medical Oncology, Dana-Farber Cancer Institute, Boston, Massachusetts, USA; 2 Department of Biostatistics and Bioinformatics, Roswell Park Cancer Institute, Buffalo, New York, USA; 3 Perlmutter Cancer Center, NYU Langone Health, New York, New York, USA

**Keywords:** tumor microenvironment, breast neoplasms, CD8-positive T-lymphocytes, inflammation, lymphocytes, tumor-infiltrating

## Abstract

**Background:**

Immune checkpoint inhibitors (ICIs) have had a profound impact on the treatment of many tumors; however, their effectiveness against triple-negative breast cancers (TNBCs) has been limited. One factor limiting responsiveness of TNBCs to ICIs is a lack of functional tumor-infiltrating lymphocytes (TILs) in ‘non-inflamed’ or ‘cold’ tumor immune microenvironments (TIMEs), although by unknown mechanisms. Targeting MUC1-C in a mouse transgenic TNBC tumor model increases cytotoxic tumor-infiltrating CD8+ T cells (CTLs), supporting a role for MUC1-C in immune evasion. The basis for these findings and whether they extend to human TNBCs are not known.

**Methods:**

Human TNBC cells silenced for MUC1-C using short hairpin RNAs (shRNAs) were analyzed for the effects of MUC1-C on global transcriptional profiles. Differential expression and rank order analysis was used for gene set enrichment analysis (GSEA). Gene expression was confirmed by quantitative reverse-transcription PCR and immunoblotting. The The Cancer Genome Atlas Breast Invasive Carcinoma (TCGA-BRCA) and Molecular Taxonomy of Breast Cancer International Consortium (METABRIC) datasets were analyzed for effects of MUC1 on GSEA, cell-type enrichment, and tumor immune dysfunction and exclusion. Single-cell scRNA-seq datasets of TNBC samples were analyzed for normalized expression associations between MUC1 and selected genes within tumor cells.

**Results:**

Our results demonstrate that MUC1-C is a master regulator of the TNBC transcriptome and that MUC1-C-induced gene expression is driven by STAT1 and IRF1. We found that MUC1-C activates the inflammatory interferon (IFN)-γ-driven JAK1→STAT1→IRF1 pathway and induces the IDO1 and COX2/PTGS2 effectors, which play key roles in immunosuppression. Involvement of MUC1-C in activating the immunosuppressive IFN-γ pathway was extended by analysis of human bulk and scRNA-seq datasets. We further demonstrate that MUC1 associates with the depletion and dysfunction of CD8+ T cells in the TNBC TIME.

**Conclusions:**

These findings demonstrate that MUC1-C integrates activation of the immunosuppressive IFN-γ pathway with depletion of TILs in the TNBC TIME and provide support for MUC1-C as a potential target for improving TNBC treatment alone and in combination with ICIs. Of translational significance, MUC1-C is a druggable target with chimeric antigen receptor (CAR) T cells, antibody-drug conjugates (ADCs) and a functional inhibitor that are under clinical development.

## Background

Triple-negative breast cancer (TNBC) is an aggressive disease with limited therapeutic options.^1^ Patients with TNBC are typically treated with neoadjuvant or adjuvant chemotherapy.^1^ Improved survival in the response of TNBCs to chemotherapy has been associated with the presence of tumor-infiltrating lymphocytes (TILs).^2 3^ Increased TILs, specifically CD8+ T cells, in the tumor immune microenvironment (TIME) are thus predictive of response to therapy and improved outcomes for patients with early-stage TNBC.^4–6^ The presence of TILs has also been linked to (1) programmed death ligand 1 (PD-L1) expression in TNBCs^7 8^ and (2) responsiveness to immune checkpoint inhibitors (ICIs), such as pembrolizumab and atezolizumab, supporting the notion that ‘non-inflamed’ or ‘cold’ TIMEs confer resistance to these agents.^9–11^ TIL density has not been significantly associated with *BRCA1/2* mutation or homologous recombination deficiency status,^12^ suggesting that recruitment of TILs may be independent of mutational burden. These findings have emphasized the need, at least in part, for identifying intrinsic TNBC cell effectors that contribute to depletion or dysfunction of TILs and thereby cold TIMEs.

MUC1-C is an oncogenic protein that is aberrantly expressed in TNBC cells.^13 14^ MUC1-C drives lineage plasticity in the progression of TNBC cells by inducing the epithelial–mesenchymal transition (EMT), epigenetic reprogramming, stemness, self-renewal capacity and drug resistance.^14^ EMT and the cancer stem cell state have been linked to immune evasion, although by unclear mechanisms.^15–17^ Along these lines, MUC1-C integrates lineage plasticity with induction of the *CD274/PD-L1* gene in human TNBC cells.^18^ Studies in a genetically engineered TNBC mouse model further showed that targeting MUC1-C with the GO-203 inhibitor, which blocks MUC1-C homodimerization and function,^13 14^ is associated with (1) suppression of PD-L1 expression, (2) increases in tumor-infiltrating CD8+ TILs and (3) induction of cognate CD8+ T cell (CTL) activity against TNBC cells.^18^ Targeting MUC1-C in this model, which is resistant to anti-PD-L1 treatment, was associated with antitumor activity.^18^ These findings supported involvement of MUC1-C in promoting immune evasion. However, there is no known association between MUC1-C and the presence of TILs in TNBC TIMEs.

The present studies investigating human TNBC cells silenced for MUC1 using tet-inducible shRNAs demonstrate that MUC1-C drives the (1) inflammatory interferon (IFN)-γ→JAK1→STAT1 pathway, (2) downstream IRF1 transcription factor, and (3) IDO1 and COX2/PTGES immunosuppressive effectors. Analysis of bulk and single TNBC cell RNA-seq datasets further showed that MUC1 associates with intrinsic expression of JAK1, STAT1, IRF1, IDO1 and PTGES. Of functional importance, MUC1 associates with depletion and dysfunction of CD8+ T cells in the TNBC TIME. These findings provide new insights into the involvement of MUC1-C in integrating lineage plasticity and immunosuppression in TNBCs.

## Methods

### Cell culture

Human BT-549 (American Type Culture Collection (ATCC)) cells were cultured in RPMI1640 medium (Thermo Fisher Scientific, Waltham, Massachusetts, USA) containing 10% fetal bovine serum (FBS; GEMINI Bio-Products, West Sacramento, California, USA), 100 µg/mL streptomycin, 100 U/mL penicillin and 10 µg/mL insulin. SUM149 (ATCC) cells were grown in Ham’s F-12 medium (Corning, Manassas, Virginia, USA) supplemented with 10 mM HEPES, 5% FBS, 100 µg/mL streptomycin, 100 U/mL penicillin, 5 µg/mL insulin and 1 µg/mL hydrocortisone. MDA-MB-468 (ATCC) cells were cultured in DMEM medium (Thermo Fisher Scientific) with 10% FBS, 100 µg/mL streptomycin, and 100 U/mL penicillin. Cells were treated with 5 µM GO-203.^14^ Cell authentication was performed by short tandem repeat analysis. Cells were monitored for mycoplasma contamination using the MycoAlert Mycoplasma Detection Kit (Lonza, Rockland, Massachusetts, USA).

### Tetracycline-inducible gene silencing

MUC1shRNA (MISSION shRNA TRCN0000122938; Sigma, St. Louis, Missouri, USA) and a control scrambled shRNA (Sigma) were inserted into pLKO-tet-puro (Plasmid #21915; Addgene, Cambridge, Massachusetts, USA). The viral vectors were produced in 293 T cells. Cells transduced with the vectors were selected for growth in 2 µg/mL puromycin. Cells were treated with 0.1% dimethyl sulfoxide (DMSO) as the vehicle control or 500 ng/mL doxycycline (DOX, Millipore Sigma).

### Real-time quantitative reverse-transcription PCR (qRT-PCR)

Total RNA was isolated using Trizol reagent (Invitrogen, Carlsbad, California, USA). cDNAs were synthesized using the High Capacity cDNA Reverse Transcription Kit (Applied Biosystems, Grand Island, New York, USA). Samples were amplified using the Power SYBR Green PCR Master Mix (Applied Biosystems) and the CFX96 Touch Real-Time PCR Detection System (Bio-Rad Laboratories, Hercules, California, USA). Primers used for qRT-PCR analysis are listed in online supplemental table S1.

10.1136/jitc-2020-002115.supp1 Supplementary data

### Immunoblot analysis

Whole-cell lysates were prepared in radioimmunoprecipitation assay (RIPA) buffer containing protease inhibitor cocktail (Thermo Fisher Scientific). Immunoblotting was performed with anti-MUC1-C (#16564, 1:1000 dilution; Cell Signaling Technology (CST), Danvers, Massachusetts, USA), anti-STAT1 (9172S, 1:1000 dilution; CST), anti-STAT1 phosphorylation (pSTAT1) (Y701) (#7167S, 1:1000 dilution; CST), anti-JAK1 (#3332S, 1:1000 dilution; CST), anti-IFN-γ (#8455S, 1:1000 dilution; CST), anti-IRF1 (#8478S, 1:1000 dilution; CST), anti-IDO1 (#86 630S, 1:1000 dilution; CST), anti-COX2 (#12 282S, 1:1000 dilution; CST), anti-prostaglandin E synthase (PTGES)(#ab62050, 1:1000 dilution; Abcam, Cambridge, Massachusetts, USA), anti-glyceraldehyde-3-phosphate dehydrogenase (GAPDH) (#5174S, 1:1000 dilution; CST) and anti-β-actin (A5441, 1:100 000 dilution; Sigma).

### RNA-seq analysis

Total RNA from cells cultured in triplicates was isolated using RNeasy Plus Mini Kit (QIAGEN). TruSeq Stranded messenger RNA (mRNA) (Illumina, San Diego, California, USA) was used for library preparation. Raw sequencing reads were aligned to the human genome (GRCh38.74) using STAR^19^ (20.1×10^6^ uniquely mapped reads per sample). Raw feature counts were normalized and differential expression analysis was carried out using DESeq2.^20^ Differential expression rank order was used for subsequent gene set enrichment analysis (GSEA), performed using the clusterProfiler package in R. Gene sets queried included the Hallmark, Canonical pathways, and GO Biological Processes Ontology collections available through the Molecular Signatures Database (MSigDB). Transcriptional regulator and motif enrichment analyses were performed using epigenetic Landscape in Silico deletion Analysis (LISA).^21^

### Statistical analysis

Each experiment was repeated at least three times. Data are expressed as the mean±SD. Unpaired Student’s t-test or Wilcoxon rank-sum test were used to examine differences between means of the two groups. A p value of <0.05 was considered a statistically significant difference.

### Analysis of publicly available cohort data

TCGA-BRCA expression and clinical annotations were obtained from the Genomic Data Commons data portal and processed via TCGAbiolinks package in R using TCGAWorkflow guided practices.^22^ Normalized METABRIC expression and clinical annotations were obtained directly from cBioPortal. Differential expression associated with MUC1 expression (MUC1-high=top quartile, MUC1-low=bottom quartile) within each respective cohort was determined by TCGAbiolinks/edgeR or limma. GSEA of differential expression was assessed using the clusterProfiler package. Queried gene sets derived from the Hallmark, Canonical pathways, and GO Biological Processes Ontology collections were retrieved from the MSigDB. Cell-type enrichment within each sample was estimated from bulk expression via xCell^23^ and TIP^24^ analyses. TNBC TCGA-BRCA and TNBC METABRIC datasets were analyzed using the tumor immune dysfunction and exclusion (TIDE) computational method to model T-cell dysfunction and T-cell exclusion.^25^ The Dysfunction Score was calculated to stratify tumors by ‘high’ and ‘low’ T-cell dysfunction and to perform differentially expressed gene (DEG) analysis. GSEA of differential expression was performed on dysfunction-associated groups/ranked DEGs.

### Analysis of publicly available TNBC scRNA-seq datasets

Data for two publicly available scRNA-seq datasets of TNBC samples (GSE75688^26^ and GSE118390^27^) were obtained directly from GEO via *GEOquery*. GSE75688 included primary tumor samples from a total of 11 breast cancer (BC) patients, including four with TNBC. Expression (transcripts per kilobase million (TPM)) was obtained, with tumor cells identified using previously determined criteria from the original publication. uniform manifold approximation and projection (UMAP) representations of remaining tumor cells were produced from total expression profiles, with expression (TPM) compared between given factors. GSE118390 included 6 patients with TNBC.^27^ Generated census counts were obtained from GEO. Low quality cells previously determined were eliminated prior to downstream analysis. Remaining cells were analyzed by Seurat.^28^ Normalization and variance stabilization were conducted using regularized negative binomial regression (sctransform).^29^ Postnormalized counts were examined, and any cells with total counts plus/minus mean average deviations were removed, leaving the final cell pool analyzed (n=870). Graph-based nearest-neighbor clustering was implemented to identify final clusters, and UMAP was applied to obtain low-dimensional representation of cells. To identify putative tumor cells from infiltrating immune cells, singleR,^30^ was employed which compares expression profiles of each individual cell to known cell-type expression profiles available within the human primary cell atlas (HPCA). Putative tumor cell clusters were selected as those that annotated >50% to epithelial cells signatures defined in HPCA. Normalized expression associations between MUC1 and select factors within tumor cells (n=515) were examined by Pearson correlation analysis.

### Data and software availability

The accession number for the RNA-seq data reported in this paper is GEO ACCESSION GSE164141

## Results

### MUC1-C activates a global transcriptional program enriched for the IFN-γ→JAK1→STAT1 signaling pathway

**Figure 1:**
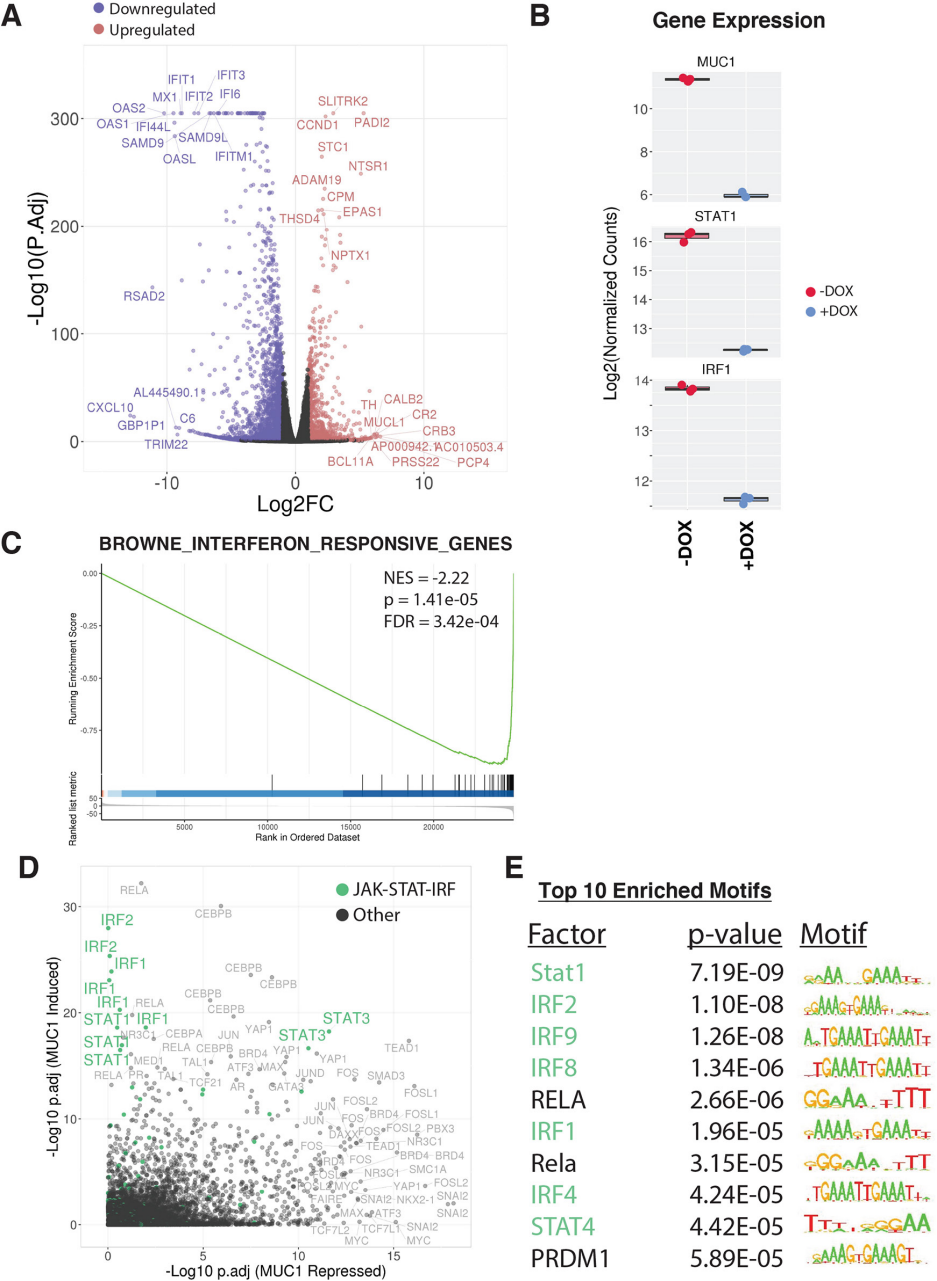
MUC1-C drives a global transcriptional program enriched for IFN signaling through STAT/IRF regulation. RNA-seq was performed on BT-549/tet-MUC1shRNA cells treated with vehicle or DOX for 7 days. (A) Volcano plot depicting DEGs identified as downregulated (blue) and upregulated (red) (FC>2, FDR<0.05) on MUC1 silencing (+DOX). Top 10 ranked up and downregulated DEGs by significance and magnitude are marked. (B) Candidate expression of STAT-IRF factors in MUC1 silenced (+DOX) cells. (C) Candidate pathway enrichment plot for the IFN regulated gene set. (D) epigenetic Landscape in Silico deletion Analysis (LISA) was applied to the top 500 downregulated (MUC1 induced) and upregulated (MUC1 repressed) DEGs. Each data point represents the significance level of regulatory potential of a given transcriptional factor derived from a unique ChIP-seq dataset. JAK-STAT-IRF factors are highlighted in green. (E) The top 10 significantly enriched motifs observed at MUC1 induced degs. DEG, differentially expressed gene; DOX, doxycycline; IFN, interferon; NES, normalized enrichment scores.

**Figure 2:**
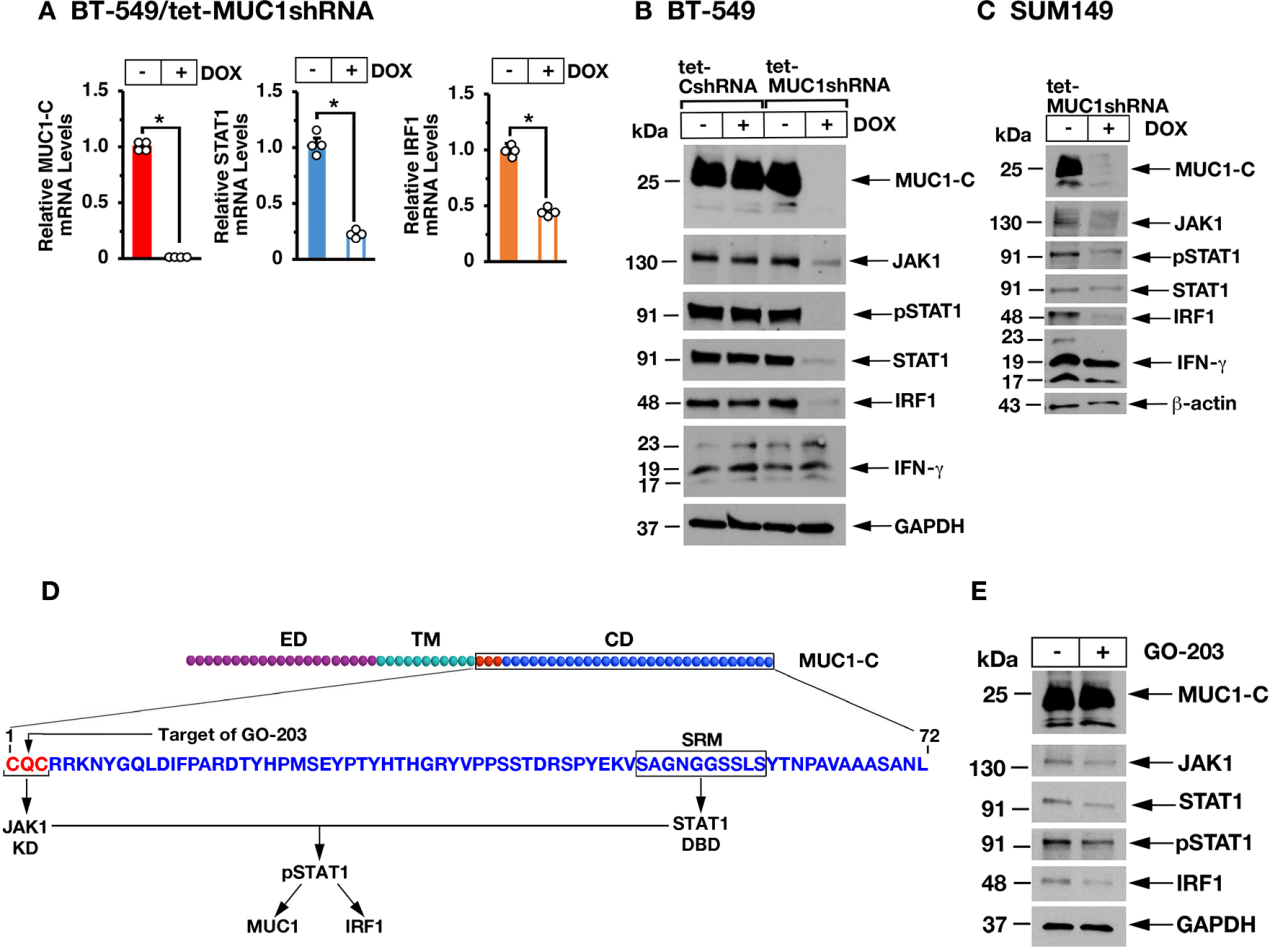
Targeting MUC1-C suppresses the JAK1→STAT1→IRF1 pathway. (A) BT-549 cells expressing a tet-MUC1 shRNA were treated with vehicle or DOX for 7 days. MUC1-C, STAT1 and IRF1 mRNA levels were analyzed by qRT-PCR. The results (mean±SD of four determinations) are expressed as relative mRNA levels compared with that obtained for vehicle-treated cells (assigned a value of 1). The asterisk (*) denotes a p value of <0.05. (B) BT-549 cells expressing a tet-control scrambled shRNA or tet-MUC1shRNA were treated with vehicle or DOX for 7 days. Lysates were immunoblotted with antibodies against the indicated proteins. (C) SUM149 cells expressing a tet-MUC1shRNA were treated with vehicle or DOX for 7 days. Lysates were immunoblotted with antibodies against the indicated proteins. (D) Schema of the MUC1-C subunit containing the 58 AA ED, the 28 AA TM and the 72 AA CD, which includes a CQC motif that binds directly to the JAK1 kinase domain. The CD also includes an SRM that interacts directly with the STAT1 DBD.^32^ MUC1-C thereby promotes the formation of JAK1 complexes with STAT1 and JAK1-mediated activation of pSTAT1.^32^ The CQC motif is the target of the GO-203 inhibitor that blocks MUC1-C homodimerization and heterodimerization with effectors, such as JAK1. (E) Lysates from BT-549 cells treated with 5 µM GO-203 for 48 hours were immunoblotted with antibodies against the indicated proteins. CD, cytoplasmic domain; DBD, DNA-binding domain; DOX, doxycycline; ED, extracellular domain; IFN, interferon; qRT-PCR, quantitative reverse-transcription PCR; SRM, serine-rich motif; TM, transmembrane domain.

Analysis of global transcriptional profiles from BT-549 TNBC cells demonstrated that inducible MUC1-C silencing results in broad changes in gene expression (3422 DEGs; false discovery rate (FDR)<0.05, fold change (FC)>2), with 1122 upregulated (MUC1-C repressed) and 2300 downregulated (MUC1-C induced) (figure 1A). Assessment of the top affected pathways revealed strong associations of MUC1-C with STAT and IFN regulated gene sets (online supplemental figure S1). Consistent with those observations, we found that silencing MUC1-C results in downregulation of (1) STAT1 and IRF1 (figure 1B) and (2) IFN pathway-responsive genes (figure 1C). To reveal master transcriptional regulators of the MUC1-C-mediated expression patterns, we applied LISA analysis,^21^ which infers regulatory potential from integrating thousands of published ChIP-seq datasets and motifs. The top master regulators associated with MUC1-C-induced expression patterns included STAT1 and IRF1, confirming that MUC1-C mediates signaling through STAT-IRF transcription factor activity (figure 1D). Motif assessment further validated that MUC1-C-induced DEGs were highly enriched for STAT and IRF motifs (figure 1E). Together, these findings implicate STAT-IRF factors as major drivers of MUC1-C-induced transcriptional responses in TNBC cells.

### MUC1-C activates JAK1→STAT1→IRF1 signaling in TNBC cells

JAK1**→**pSTAT1 signaling activates the primary response *IRF1* gene, which encodes a transcription factor that drives downstream IFN-γ pathway target genes.^31^ Consistent with the observations from RNA-seq analysis, qRT-PCR confirmed that silencing MUC1-C in BT-549 TNBC cells results in downregulation of STAT1 and IRF1 transcripts (figure 2A). Silencing MUC1-C also resulted in suppression of JAK1, pSTAT1, STAT1 and IRF1 proteins (figure 2B) and had little, if any, effect on intrinsic IFN-γ expression (figure 2B). Studies in SUM149 TNBC cells demonstrated similar effects with MUC1-C silencing, indicating that the results are not limited to a single TNBC cell line (figure 2C). MUC1-C includes a 72 aa cytoplasmic domain that directly interacts with diverse effectors,^13 14^ including the JAK1 kinase domain and STAT1 DNA-binding domain (figure 2D), with induction of JAK1-mediated pSTAT1.^32^ Treatment with the GO-203 inhibitor,^13 14^ which blocks the MUC1-C CQC motif that binds to JAK1 (32), decreased JAK1, pSTAT1 and IRF1 expression (figure 2E), supporting a role for MUC1-C in driving the intrinsic IFN-γ pathway in TNBC cells.

### Targeting MUC1-C suppresses IDO1 and COX2/PTGES

**Figure 3:**
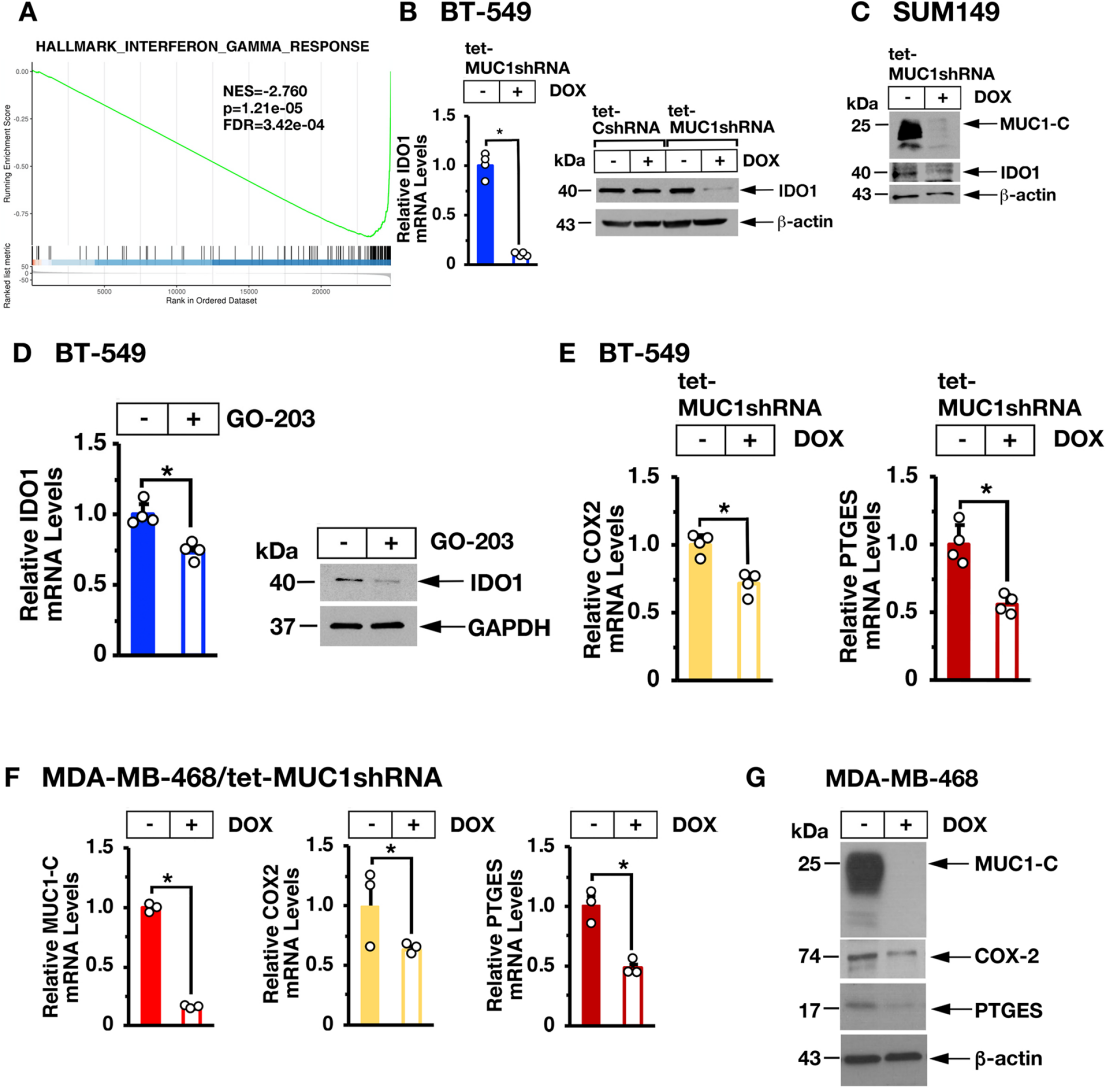
Targeting MUC1-C suppresses IDO1 and COX2. (A) Candidate pathway enrichment plot for the hallmark IFN-γ response derived from RNA-seq on BT-549/tet-MUC1shRNA cells treated with vehicle or DOX for 7 days. (B) The indicated BT-549 cells were treated with vehicle or DOX for 7 days. IDO1 mRNA levels were analyzed by qRT-PCR (left). The results (mean±SD of four determinations) are expressed as relative mRNA levels compared with that obtained for vehicle-treated cells (assigned a value of 1). Lysates were immunoblotted with antibodies against the indicated proteins (right). (C) SUM149/tet-MUC1shRNA cells were treated with vehicle or DOX for 7 days. Lysates were immunoblotted with antibodies against the indicated proteins. (D) BT-549 cells left untreated or treated with 5 µM GO-203 for 48 hours were analyzed for IDO1 mRNA levels by qRT-PCR (left). The results (mean±SD of four determinations) are expressed as relative mRNA levels compared with that obtained for untreated cells (assigned a value of 1). Lysates were immunoblotted with antibodies against the indicated proteins (right). (E, F) BT-549/tet-MUC1shRNA (E) and MDA-MB-468/tet-MUC1shRNA (F) cells treated with vehicle or DOX for 7 days were analyzed for COX2/PTGS2 and PTGES mRNA levels by qRT-PCR. The results (mean±SD of three determinations) are expressed as relative mRNA levels compared with that obtained for vehicle-treated cells (assigned a value of 1). (G) Lysates from MDA-MB-468/tet-MUC1shRNA cells treated with vehicle or DOX for 7 days were immunoblotted with antibodies against the indicated proteins. DOX, doxycycline; IFN, interferon; qRT-PCR, quantitative reverse-transcription PCR.

In concert with these findings, global expression analysis demonstrated that silencing MUC1-C is significantly associated with suppression of the IFN-γ pathway (figure 3A and online supplemental figure S2). From these observations, we identified potential MUC1-C-driven genes linked to IFN-γ signaling, IRF1-mediated transcription and immune suppression. Among these, we found that silencing MUC1-C in BT-549 (figure 3B, left and right) and SUM149 (figure 3C) cells is associated with downregulation of IDO1, which is activated by IRF1 and plays a key role in immunosuppression.^33^ Targeting MUC1-C with the GO-203 inhibitor further confirmed that MUC1-C drives IDO1 expression (figure 3D, left and right). In addition, we found that MUC1-C drives expression of COX2/PTGS2 and PTGES (figure 3E, left and right) that together synthesize PGE2, an inhibitor of T-cell function^34^ associated with decreased survival in TNBC tumors.^35 36^ In support of these results, studies of MDA-MB-468 TNBC cells confirmed that MUC1-C drives COX2 and PTGES expression (figure 3F, G).

### MUC1 associates with depletion of immune cell infiltration

**Figure 4:**
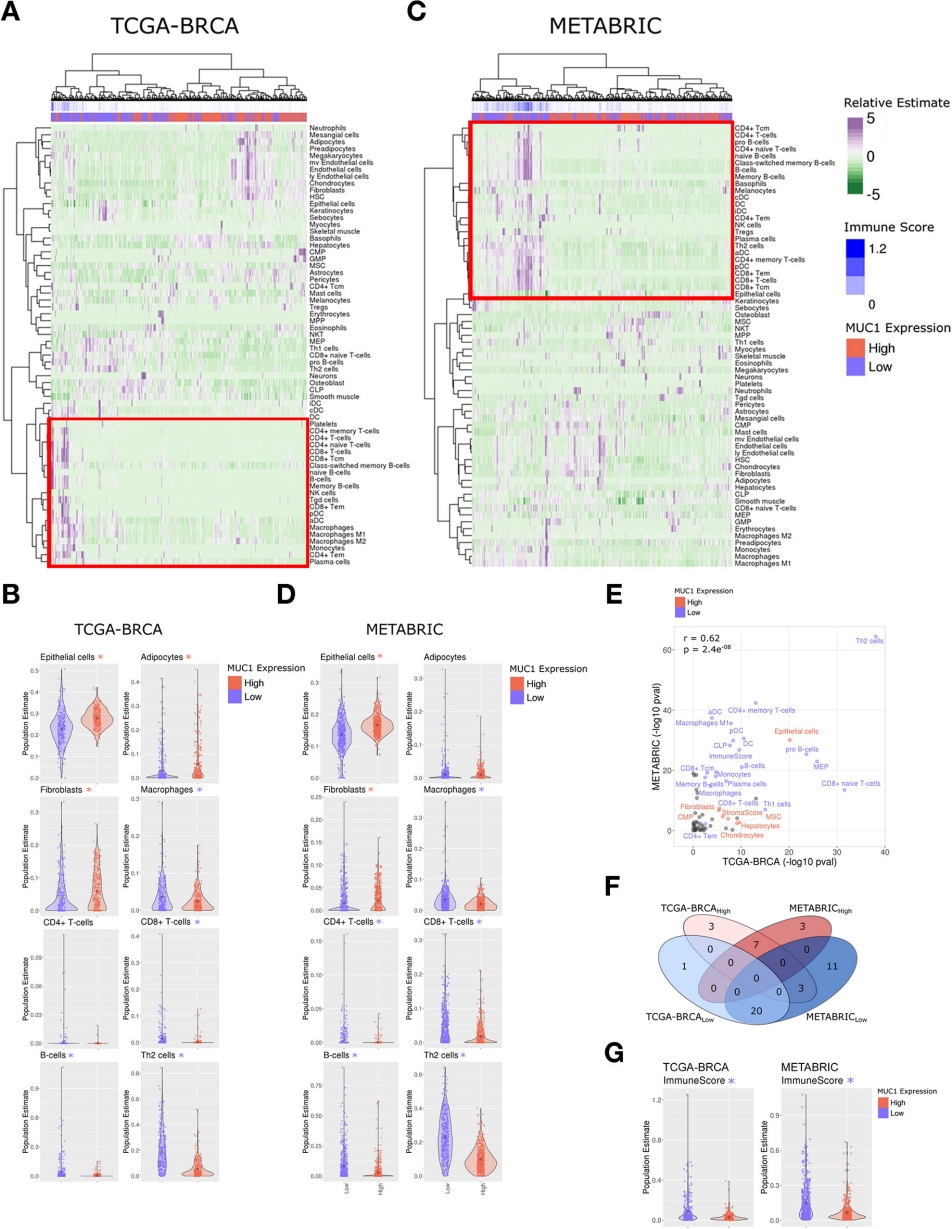
MUC1-high expressing tumors in the TCGA-BRCA and METABRIC cohorts associate with immune cell depletion. (A) Heatmap depicting cell-type enrichment analysis (xCell) in MUC1-high and MUC1-low expressing tumors from the TCGA-BRCA cohort. (B) Select cell-type enrichments determined between MUC1-high and MUC1-low tumors from the TCGA-BRCA cohort. The asterisk represents significant difference (Wilcox rank-sum test, p<0.05) between groups. The color of the asterisk represents the enrichment group (red indicates MUC1 high, blue indicates MUC1 low). (C, D) Heatmap and candidate cell-type estimates from the xCell analysis of METABRIC cohort. (E) Comparison of MUC1 associated cell-type enrichments observed between TCGA-BRCA and METABRIC cohorts. Highlighted cell types represent those significantly associated with MUC1-high (red) and MUC1-low (blue) in both cohorts. Pearson correlation is shown. (F) Overlap of significantly altered MUC1 associated cell types identified between TCGA-BRCA and METABRIC cohorts. (G) The aggregate immune score derived from xCell analysis between MUC1-high and MUC1-low tumors in TCGA-BRCA (left) and METABRIC (right) cohorts. TNBC, triple-negative breast cancer; xCell, cell-type estimation analysis.

In extending these findings to primary BC tissues, we first performed cell-type estimation analysis (xCell)^23^ of bulk tumor RNA-seq data within the TCGA-BRCA cohort (n=1082 primary tumor, 113 normal). Stratifying tumors identified BC subsets with MUC1-high and MUC1-low expression (n=271 per group), and subsequent hierarchical clustering of tumors based on cell-type estimation largely separated tumors by MUC1 status (figure 4A). Notably, the major tumor cluster enriching for MUC1-high expression was associated with decreased estimates of immune cell infiltration. Closer examination showed that MUC1-high tumors significantly associate with depletion of immune cell populations, including CD8 +T cells (Wilcox-test: p=2.02e-05), CD8 +naive T cells (p=2.82e-32), CD4 +T cells, B cells, Th2 cells and macrophages (figure 4B and online supplemental table S2). Analysis of the independent METABRIC cohort of BC tissues (n=1904) similarly revealed significant depletion of immune cell infiltrates in MUC1-high relative to MUC1-low tumors (n=476 per group), specifically CD8 +T cells (p=9.55e-19), CD8 +naive T cells (3.38e-14), B cells and Th2 cells among others (figure 4C, D and online supplemental table S3). Overall patterns of cell-type enrichment for MUC1-high and MUC1-low tumors were significantly correlated between the TCGA-BRCA and METABRIC cohorts (figure 4E, F). Moreover, quantification of total immune activity based on the integration of all immune cell-type estimates was significantly reduced in MUC1-high tumors in both cohorts (figure 4G; p=2.37e-10 and 1.81e-27 (Wilcox Test), for TCGA-BRCA and METABRIC, respectively). Secondary assessment of immune infiltration and overall immune activity was accomplished via Tracking Tumor Immunophenotype (TIP) analysis,^24^ which largely confirmed results from cell-type estimation, including suppression of T and B cell populations, as well as decreased overall immune activity, in MUC1-high as compared with MUC1-low tumors (online supplemental figure S3A–C).

### MUC1 expression in TNBC tumors associates with activation of the IFN-γ pathway and CD8+ T-cell depletion and dysfunction

**Figure 5:**
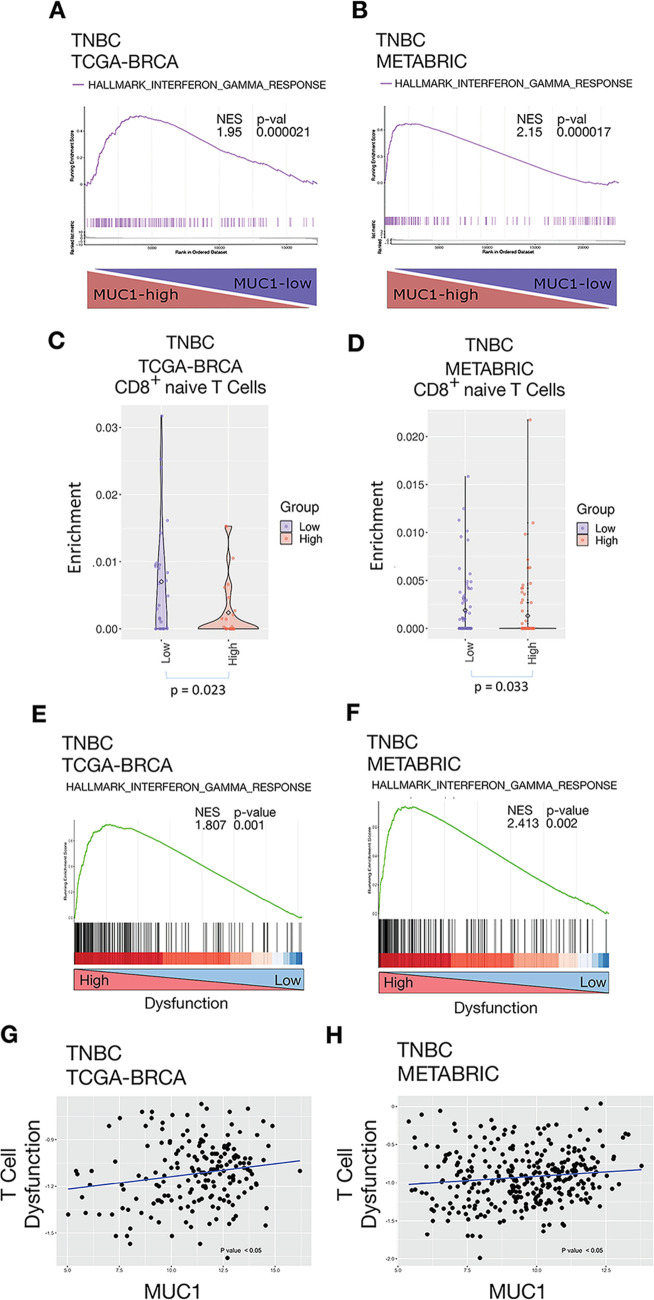
Association of MUC1 with activation of the IFN-γ pathway and CD8+ T-cell depletion and dysfunction. (A, B) Enrichment plots for the IFN-γ pathway, comparing MUC1-high to MUC1-low TNBC tumors in the TCGA-BRCA (A) and METABRIC (B) cohorts. (C, D) Comparison of MUC1-high (red) and MUC1-low (blue) associated CD8+ naïve T-cell enrichments observed for the TNBC TCGA-BRCA (C) and TNBC METABRIC (D) cohorts. Pearson correlations are shown. (E, F) Enrichment plots for the IFN-γ pathway comparing T-cell dysfunction-high to T-cell dysfunction-low TNBC tumors in the TCGA-BRCA (E) and METABRIC (F) cohorts. (G, H) Scatter plots showing correlations of MUC1 and T-cell dysfunction in TNBCs from TCGA-BRCA (G) and METABRIC (H) cohorts. IFN, interferon; TNBC, triple-negative breast cancer.

In extending these findings, we found by analysis of TNBC tumors in the TCGA-BRCA and METABRIC datasets that MUC1 associates with activation of the IFN-γ pathway (figure 5A, B) and expression of JAK1, STAT1, IRF1, IDO1 and PTGES (online supplemental figure S4A, B). In addition, we found that MUC1 expression in TNBCs from both datasets significantly associates with depletion of CD8 +naive T cells (figure 5C, D). We also analyzed the TNBC TCGA-BRCA and TNBC METABRIC datasets with the TIDE computational method to model T-cell dysfunction and T-cell exclusion.^25^ Using this method, we stratified TNBC tumors by evidence of high and low T-cell dysfunction and applied GSEA to analyze the functional enrichment of pathways within T-cell dysfunction subgroups. Notably, the IFN-γ pathway was highly enriched in TNBCs with a high T-cell dysfunction profile (figure 5E, F). Moreover, we found that MUC1 significantly correlates with T-cell dysfunction in both TNBC datasets (figure 5G, H), indicating that MUC1-induced activation of the IFN-γ pathway contributes to CD8+ T-cell exclusion and dysfunction.

### Association of MUC1 with STAT-IRF expression in TNBC single cells

**Figure 6:**
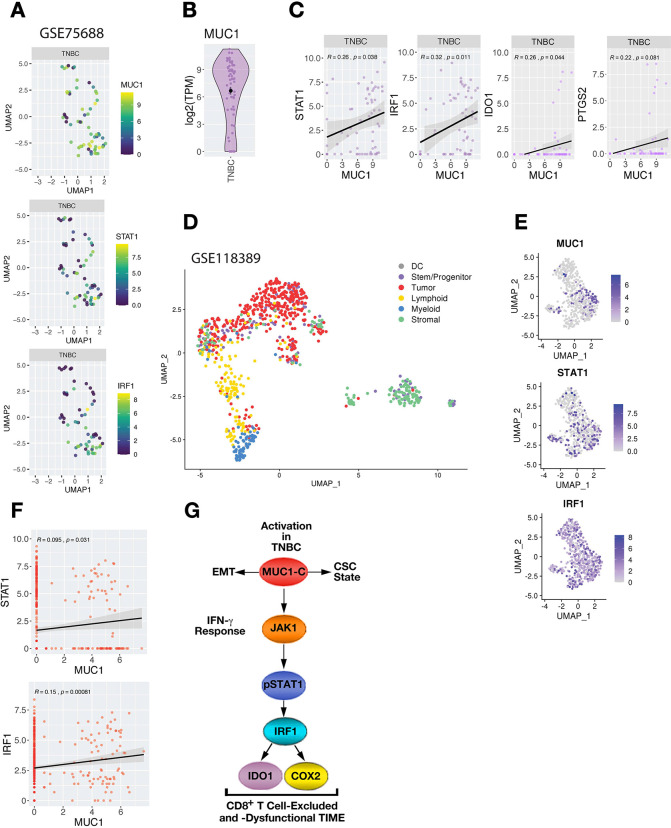
Association of MUC1 with STAT-IRF expression in single TNBC cells. (A) MUC1 expression in individual TNBC cells in GSE75688. (B) Expression of MUC1, STAT1 and IRF1 across UMAP projections of individual tumor cells identified in GSE75688. (C) Correlation of normalized MUC1 expression. (C) Expression analysis between MUC1 and with STAT1, IRF1, IDO1 and COX2/PTGS2 consistently showing positive Pearson correlations in TNBC cells (r>0.2). (D) UMAP representation of total cells analyzed from GSE118389 dataset. cell annotation analysis identified cells as either DC, stem/progenitor like cells, tumor cells (epithelial), lymphoid lineage cells (B cells, T cells, and NK cells), myeloid lineage cells (macrophage, monocytes, neutrophils, and myeloid progenitors), or stromal cells (endothelial, fibroblasts, smooth muscle, and neurons). (E) Expression of MUC1, IRF1 and STAT1 in identified tumor cells after filtering and reclustering. (F) Correlations between MUC1 and STAT1 and IRF1 expression across identified tumor cells. Pearson correlation is shown. (G) Model depicting MUC1-C-driven integration of EMT, stemness and immune evasion in TNBCs. MUC1-C drives EMT, epigenetic reprogramming and chromatin remodeling in TNBC cells.^14^ MUC1-C integrates lineage plasticity with induction of (1) the IFN-γ→JAK1→STAT1→IRF1 pathway; and (2) the downstream IDO1 and COX2 effectors, which contribute to suppression and/or dysfunction of CD8+ T cells in TNBC TIMEs. CSC, cancer stem cell; DC, dendritic cell; EMT, epithelial–mesenchymal transition; IFN, interferon; TNBC, triple-negative breast cancer.

To complement analyzes of bulk RNA-seq data in the TCGA and METABRIC cohorts and to overcome the potential confounding issues associated with complex cellular compositions, we examined relationships between MUC1 and STAT1/IRF1 expression within TNBC tumor cells analyzed by single-cell RNA-seq (scRNA-seq). We first analyzed expression data obtained in tumor cells from primary TNBCs (GSE75688; online supplemental figure S5A, B).^26^ MUC1 was commonly detected in TNBC tumor cells (figure 6A), as were STAT1 and IRF1 (figure 6B). In concert with the results from studying bulk RNA-seq data in TCGA and METABRIC cohorts, we found that MUC1 expression significantly correlates with STAT1 and IRF1 in TNBC cells (figure 6C). MUC1 also associated with IDO1 and COX2/PTGS2, consistent with driving an immunosuppressive pathway (figure 6C). We went further to analyze a second scRNA-seq dataset obtained from six primary TNBCs (GSE118390, online supplemental figure S6A).^27^ Clustering and cell annotation analyses were performed to identify and stratify tumor cells from stromal and immune cell populations within each patient (figure 6D and online supplemental figure S6B–D). Confirming previous analyses, MUC1 expression was detectable in TNBC tumor cell populations, along with STAT1 and IRF1 (figure 6E). Furthermore, MUC1 expression significantly correlated with these effectors across TNBC cells, which was not confounded by overall sequencing depth (figure 6F and online supplemental figure S6E).

## Discussion

Treatment of early-stage TNBCs with chemotherapy and ICIs has been limited by low levels of activity.^9–11^ This lack of responsiveness has been attributed in part to the absence or dysfunction of TILs in the TIME.^5 12 37 38^ Expression of MUC1-C in human TNBC cell lines has been linked to induction of PD-L1 expression (figure 6G).^14^ Additionally, studies in a mouse transgenic TNBC tumor model have demonstrated that MUC1-C (1) induces PD-L1, (2) decreases tumor-infiltrating CD8+ T cells, and (3) suppresses antitumor activity.^18^ In addressing how MUC1-C contributes to immune evasion, we found in human TNBC cells that MUC1-C drives IFN-γ→JAK1→STAT1 signaling and induction of the downstream IRF1 activator of IFN-γ–target genes (figure 6G). The IFN-γ pathway can play roles in both tumor immune surveillance and evasion.^39 40^ In support of promoting immune evasion, we found that MUC1-C activates the IFN-γ pathway-driven immunosuppressive IDO1 and COX2/PTGES genes (figure 6G). IDO1, which is upregulated in TNBCs by unclear mechanisms, reduces levels of tryptophan in the TIME that are essential for T-cell proliferation and immune function.^33^ COX2 expression is also elevated in TNBCs^36^ and, as a result, increased production of PGE2 contributes to T-cell dysfunction.^34^ Our findings that MUC1-C activates the IFN-γ→STAT1→IRF1 pathway with induction of IDO1 and COX2 provided support for the potential importance of MUC1-C in driving the exclusion and/or dysfunction of CD8+ T cells in the TNBC TIME (figure 6G).

MUC1-C is activated in the response of epithelial cells to inflammation that, if prolonged with repeated cycles of damage and repair can promote oncogenesis.^14 41^ To address the contention that MUC1-C may contribute to chronic inflammation in human tumors, we extended the findings from TNBC cell lines to studies of TNBC samples. Analysis of the BRCA-TCGA and METABRIC datasets resulted in the systematic findings that MUC1 associates with activation of the IFN-γ→STAT1→IRF1 pathway and that MUC1-high and MUC1-low expressing tumors segregate based on cell-type estimations. One striking observation was that MUC1-high tumors significantly associate with depletion of immune cell populations of CD8+ T cells, CD4+ T cells, B cells and macrophages. These findings were not restricted to exclusion of CD8+ T cells. In this regard, we also found that in that MUC1 integrates activation of the IFN-γ pathway with T-cell dysfunction, indicating that intrinsic MUC1 expression in TNBCs may promote TIMEs with both depleted and dysfunctional CTLs. Accordingly, we extended this line of investigation by digging into TNBC scRNA-seq datasets.^26 27^ Analysis of the single-cell data showed the MUC1 significantly associates with STAT1, IRF1, IDO1 and COX2, confirming the relationship between MUC1 and the immunosuppressive IFN-γ pathway. Our findings therefore link MUC1 to IFN-γ signaling that, when chronically activated in tumors, has been shown to promote a suppressive TIME.^39 42^

Our previous studies of TNBC models demonstrated that MUC1-C induces EMT and stemness (figure 6G),^14 43–45^ which promote lineage plasticity and have been linked to immune evasion.^15–17^ Those findings and the present results in TNBC tumors indicate that targeting MUC1-C could be of clinical importance in disrupting the integration of lineage plasticity with immunosuppression. Our results also support MUC1-C as a target for potentially improving responsiveness of TNBCs to immunotherapies that are limited by ‘cold’ TIMEs. Along these lines, targeting MUC1-C with the GO-203 inhibitor in a mouse TNBC model increased infiltration of functional CD8+ T cells in the TIME in association with induction of antitumor activity.^18^ GO-203 has been evaluated in phase I clinical trials with an acceptable safety profile and has been reformulated in nanoparticles to sustain drug exposure^14^ as, for instance, in combinations with ICIs that are limited in terms of effectiveness by immune cell-depleted TIMEs. An antibody targeting the MUC1-C extracellular domain has been developed as (1) an antibody–drug conjugate that is effective against human TNBC cells growing in vitro and as tumor xenografts,^14 46^ and (2) a CAR T cell that is entering clinical evaluation, both of which represent potential agents for eliminating MUC1-C-expressing TNBC tumor cells and reversing immune suppression. Finally, our findings that MUC1-C drives the IFN-γ pathway with induction of immunosuppressive factors informs additional strategies for treatment with IDO1 and/or COX2 inhibitors alone and in combination with MUC1-C-directed agents to target MUC1-C-expressing TNBCs with CD8+ T cell-depleted and/or T cell-dysfunctional TIMEs.

## Data Availability

Data are available in a public, open access repository. The accession number for the RNA-seq data reported in this paper is GEO ACCESSION GSE164141.
