# Correction: MUC1-C integrates activation of the IFN-γ pathway with suppression of the tumor immune microenvironment in triple-negative breast cancer

**DOI:** 10.1136/jitc-2020-002115corr1

**Published:** 2021-10-13

Yamashita N, Long M, Fushimi A, *et al*. MUC1-C integrates activation of the IFN-γ pathway with suppression of the tumor immune microenvironment in triple-negative breast cancer. *J Immunother Cancer.* 2021;9:e002115. doi: 10.1136/jitc-2020-002115.

This article has been corrected since it first published. The provenance and peer review statement has been added.

