# Supplementary material for: MUC1-C integrates activation of the IFN-γ pathway with suppression of the tumor immune microenvironment in triple-negative breast cancer
Source: J Immunother Cancer. 2021 Jan 24;9(1):e002115. doi: 10.1136/jitc-2020-002115 (PMC7839859; doi:10.1136/jitc-2020-002115)
Supplement: Supplementary data [file jitc-2020-002115supp001.pdf]

## Top 30 Downregulated Pathways: BT549_MUC1 shRNA

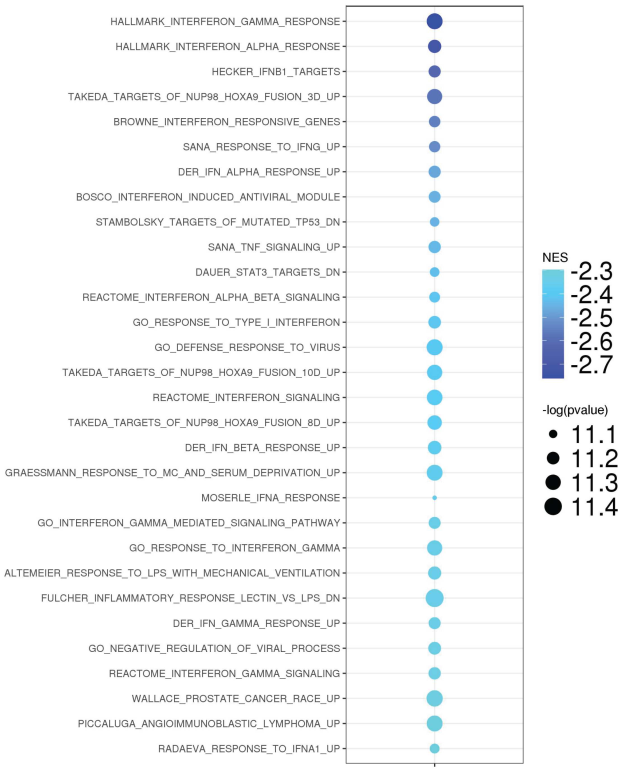


## Top 30 Upregulated Pathways: BT549_MUC1 shRNA

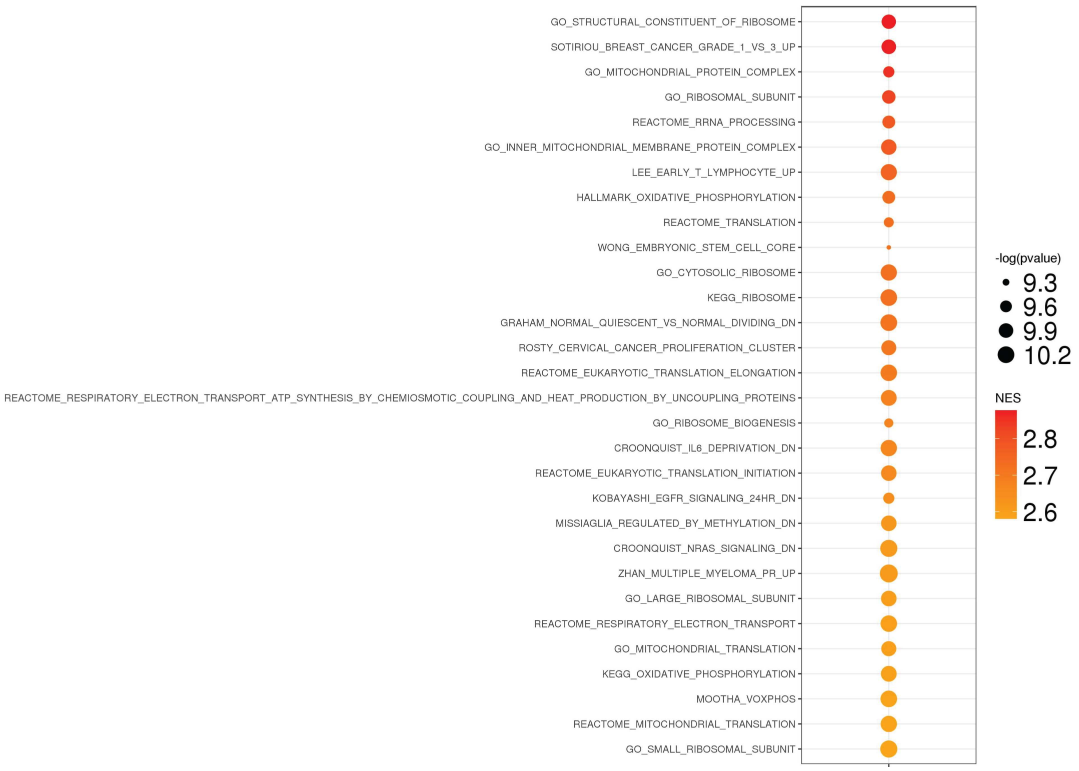


1

**Supplemental Figure S1. RNA-seq analysis of MUC1 silenced BT-549 cells.** Top 30 significantly enriched pathways activated (top) and suppressed (bottom) upon MUC1 silencing.



Yamashita N, *et al. J Immunother Cancer* 2021; 9:e002115. doi: 10.1136/jitc-2020-002115

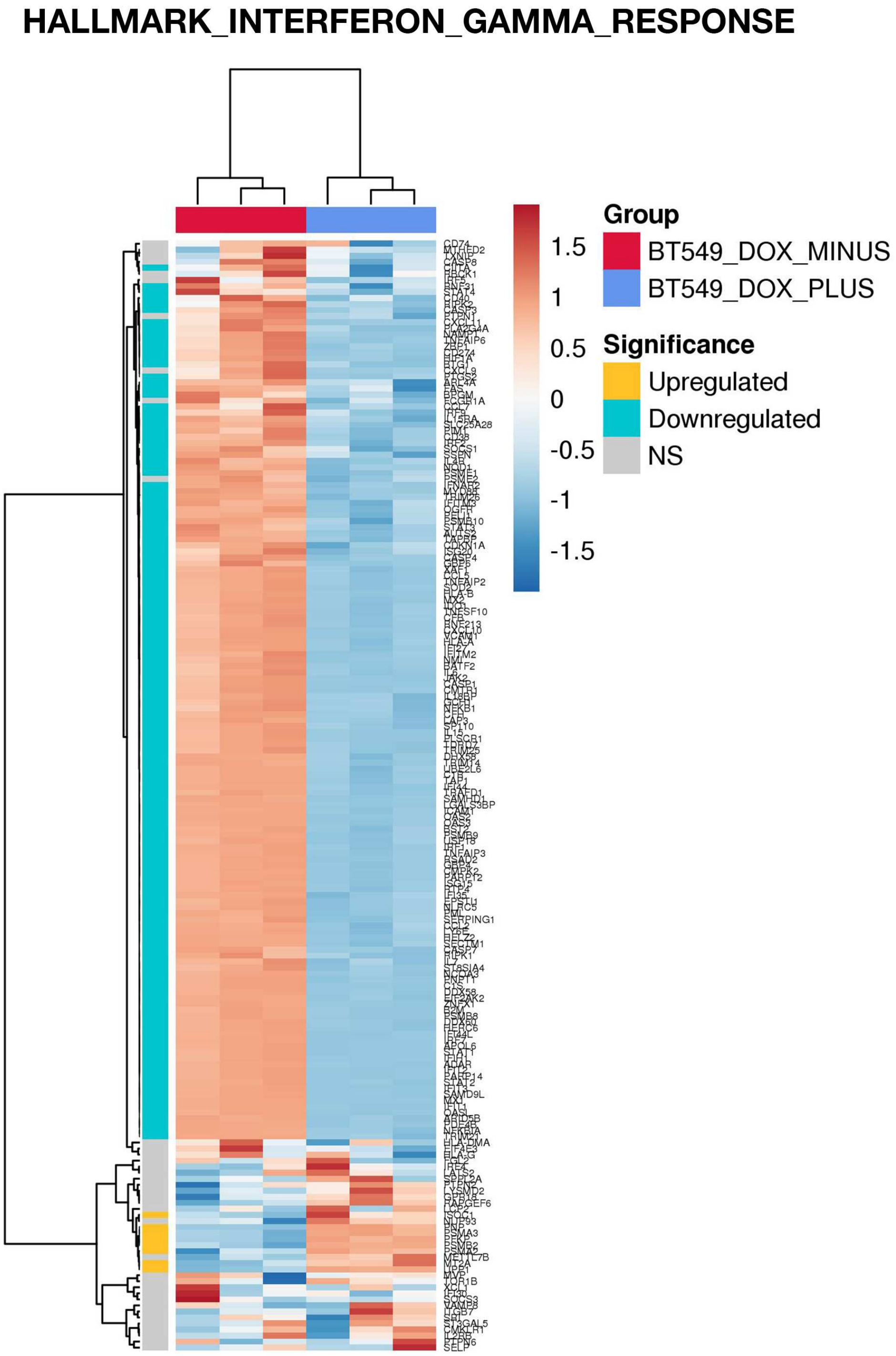


**Supplemental Figure S2. Effects of MUC1-C silencing on IFN-γ pathway genes.** Heatmap representing all genes from within the Hallmark IFN-γ Response pathway. Significant DEGs are shown as row annotations (downregulated = teal, upregulated = gold).

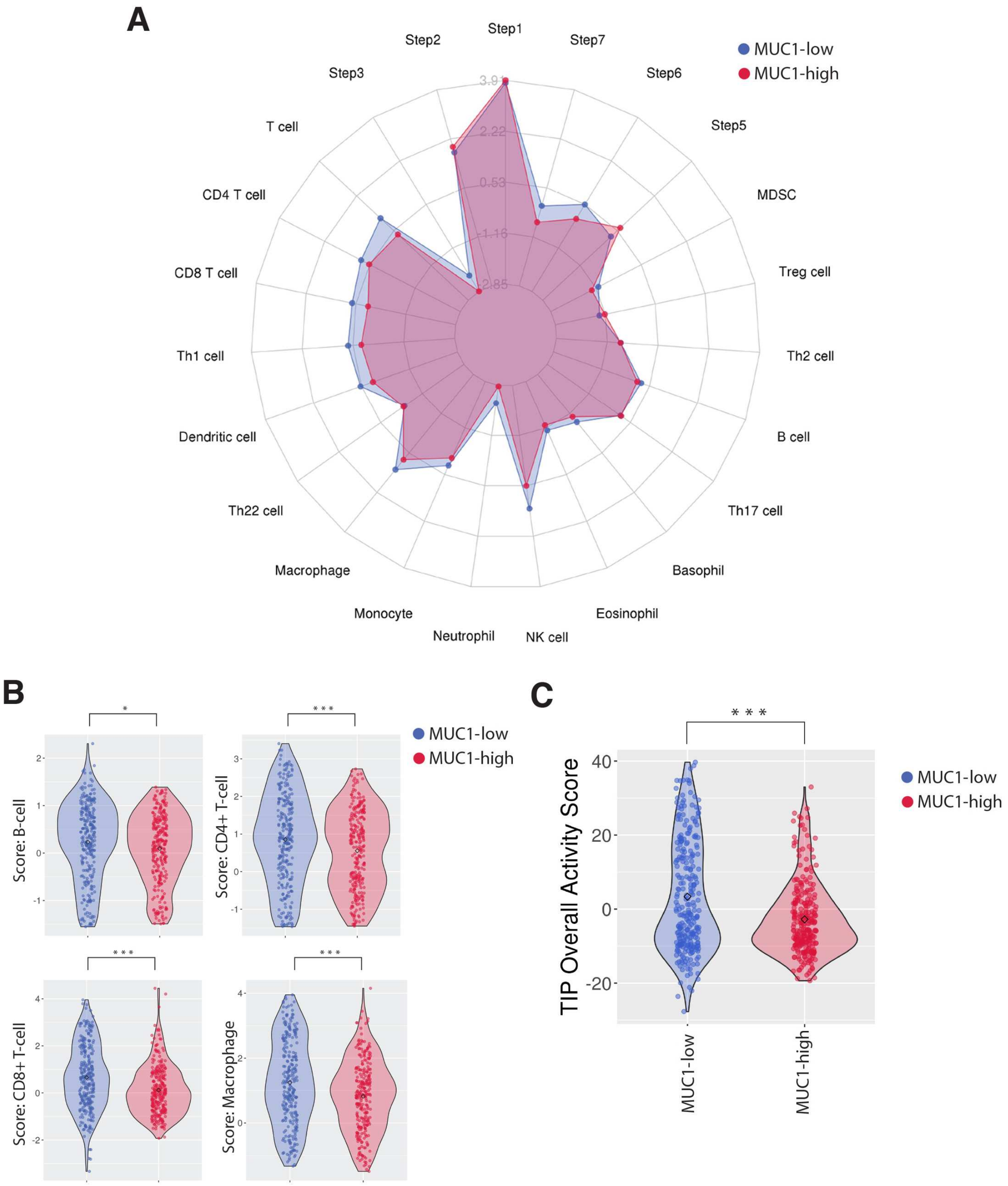


**Supplemental Figure S3. TIP assessments in MUC1-high as compared to MUC1-low tumors. A.** Radial plot showing summary of TIP analysis. Lines/points represent mean enrichment scores for each immune activation step or cell type recruitment determined for MUC1-low (blue) and MUC1-high (red) groups. **B.** Select cell type recruitment scores from TIP analysis representing significant enrichments observed between MUC1-high and MUC1-low tumors from the TCGA-BRCA cohort.



Yamashita N, *et al. J Immunother Cancer* 2021; 9:e002115. doi: 10.1136/jitc-2020-002115

Significance between MUC1-high and MUC1-low groups was determined by Wilcoxon signed-rank test (* $p < 0.05$; ** $p < 0.01$; *** $p < 0.001$). **C.** Overall immune activity determined by TIP analysis between MUC1-high and MUC1-low tumors from the TCGA-BRCA cohort.

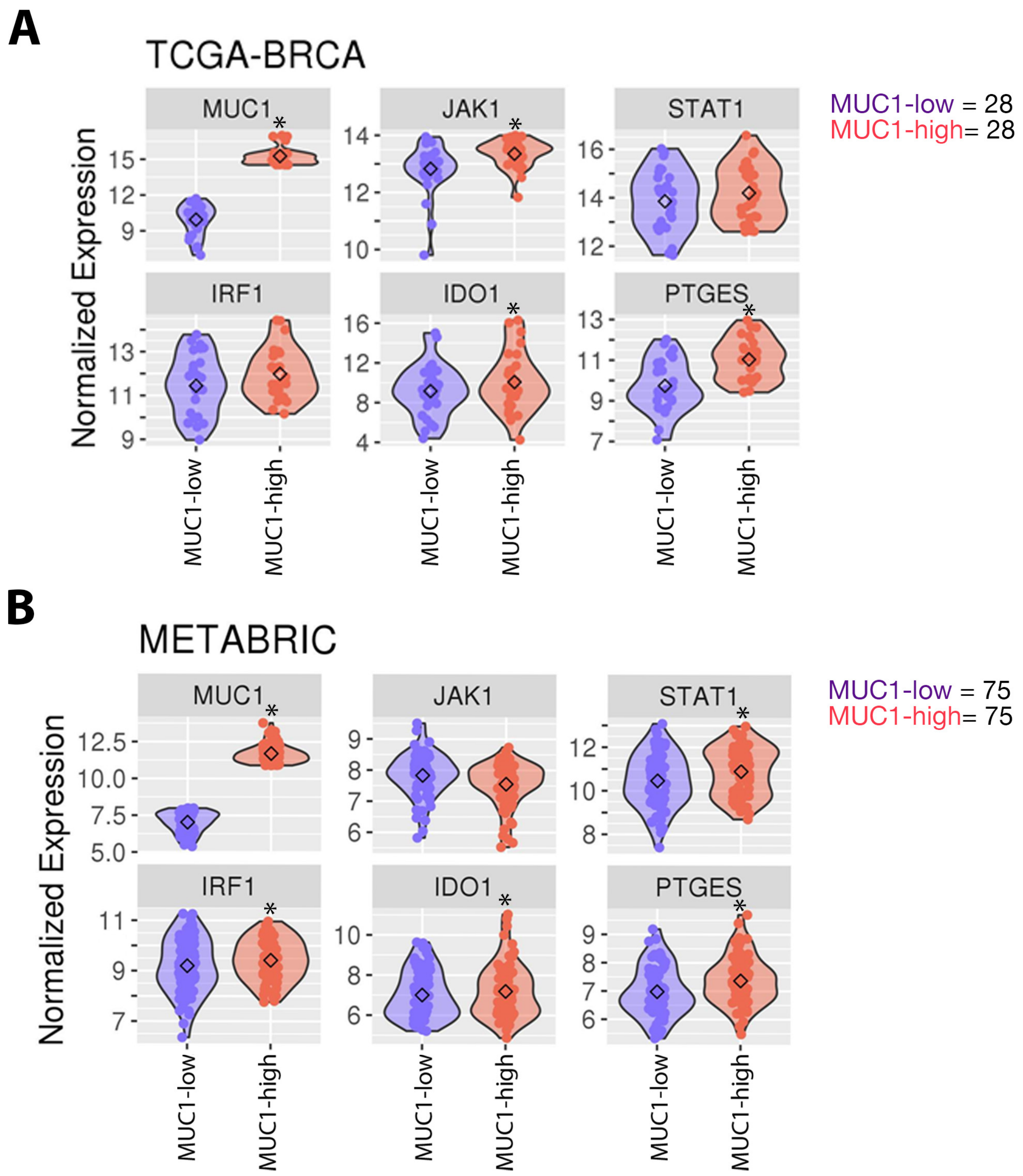


**Supplemental Figure S4. Expression of JAK1-STAT1-IRF1 and effectors IDO1 and PTGS2 in MUC1-high and MUC1-low expressing TNBC.** Normalized expression of select genes in TNBC samples obtained from TCGA-BRCA (**A**) and METABRIC (**B**) cohorts, stratified by MUC1 expression (top and bottom quartiles). Magnitude and significance of observed differences between MUC1 groups is shown (* $p < 0.05$).

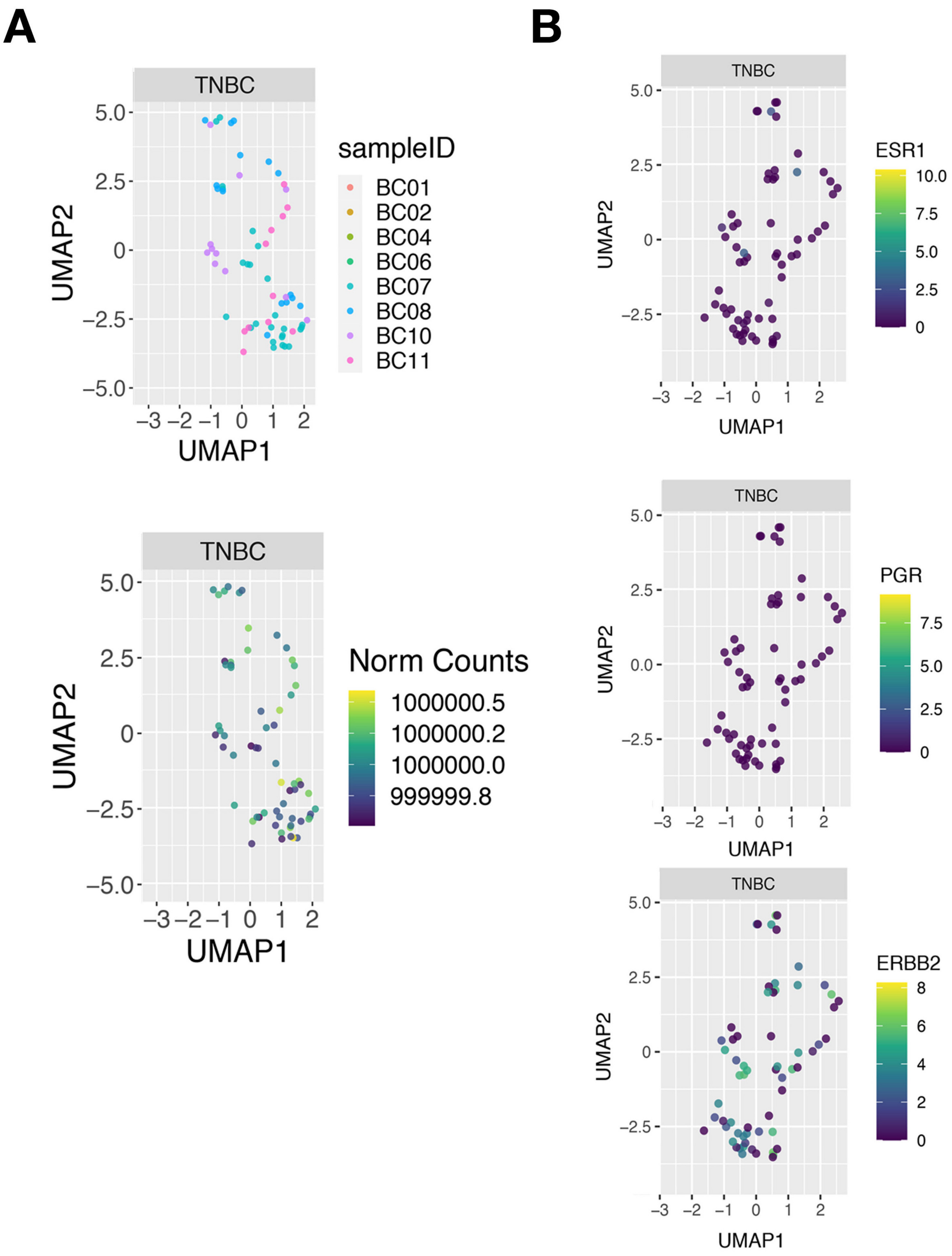


**Supplemental Figure S5. Assessment of MUC1 and inflammatory driver expression in TNBC scRNA-seq dataset GSE75688.** A. UMAP representations of TNBC tumor cells identified in GSE75688 by BC subtypes. Individual patients within each group are highlighted (upper), as well as total expression counts (TPM) mapped to individual cells (lower). B. Expression of ESR1, PGR and ERBB2 (HER2) mapped to individual tumor cells was performed to confirm TNBC subtype.



Yamashita N, *et al. J Immunother Cancer* 2021; 9:e002115. doi: 10.1136/jitc-2020-002115

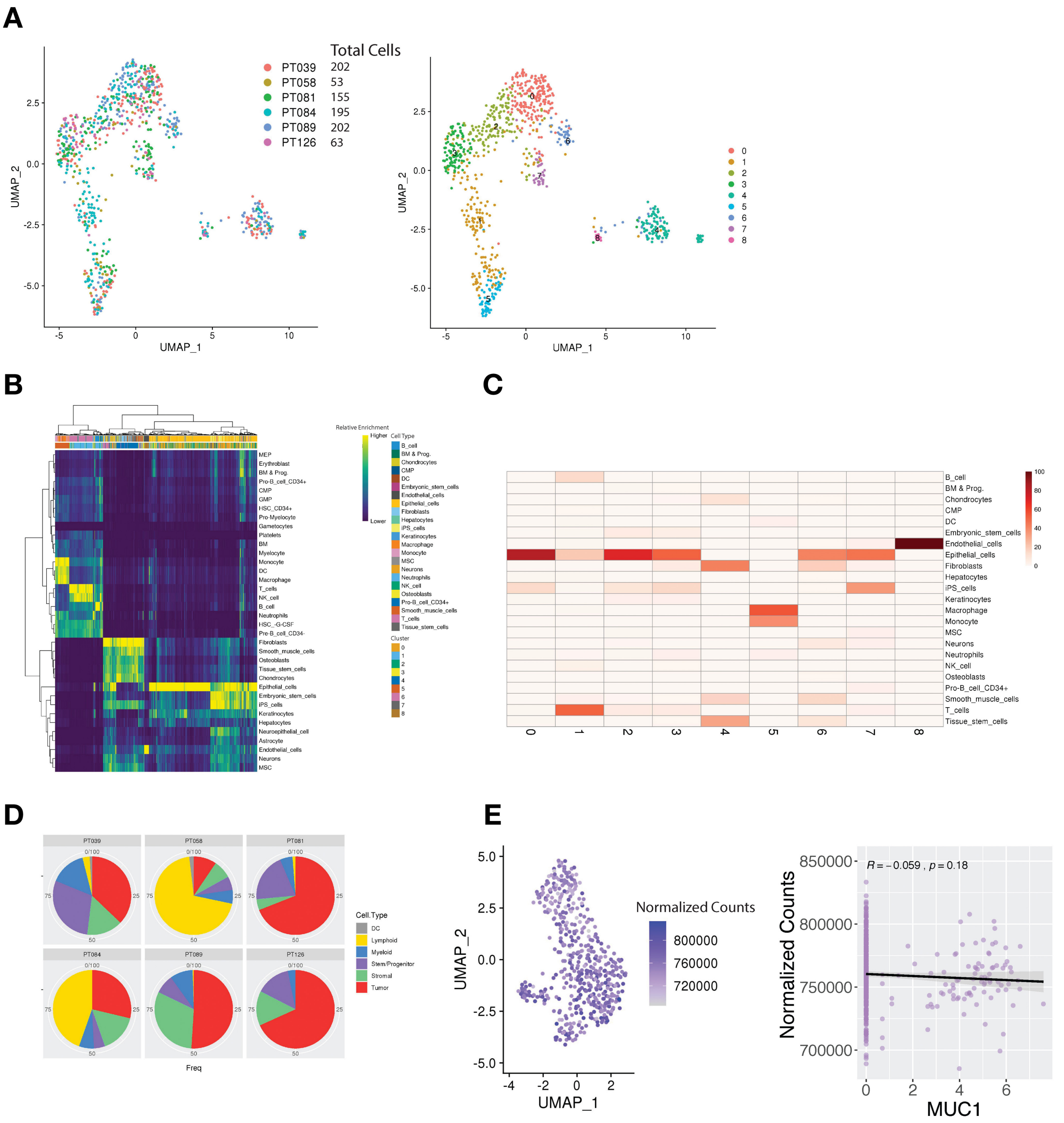


**Supplemental Figure S6. Assessment of MUC1 and inflammatory driver expression in TNBC scRNA-seq dataset GSE118389.** A. UMAP representations of total cells analyzed in GSE118389. Individual patients within each group are highlighted (left), as well identified clusters based on variable gene expression (right). B. Heatmap depicting the cell annotation scores derived from comparing each individual cell to known transcriptome references available from the human primary cell atlas (HPCA) using SingleR. C. Heatmap representing the proportions of cell types inferred within each identified cell cluster. D. Proportions of cell annotations mapping to cell types within each patient. E. UMAP representation of identified and filtered

tumor cells from within total cell populations. Total normalized counts are shown (left), as well as the relationship between MUC1 to total counts (right).

**Supplemental Table S1. Primers used for qRT-PCR analysis.**

| Primer | FWD | REV |
|---|---|---|
| MUC1-C | AGACGTCAGCGTGAGTGATG | GCCAAGGCAATGAGATAGAC |
| STAT1 | GGAACTTGATGGCCCTAAAGGA | ACAGAGCCCACTATCCGAGACA |
| IRF1 | CATGGCTGGGACATCAACAA | TTGTATCGGCCTGTGTGAATG |
| IDO1 | TCTCATTTCGTGATGGAGACT | GTGTCCCGTTCTTGCATTTGC |
| COX2 | GCTTTCACCAACGGGCTGGG | AAGACCTCCTGCCCCACAGCAA |
| PTGES | GGAAACTGCAAATGTCCCCTTGAT | CACATCTCAGGTCACGGGTCTA |
| GAPDH | CCATGGAGAAGGCTGGGG | CAAAGTTGTCATGGATGACC |



Yamashita N, *et al. J Immunother Cancer* 2021; 9:e002115. doi: 10.1136/jitc-2020-002115

**Supplemental Table S2. Analysis of MUC1 association with cell populations in the TCGA-BRCA cohort.**

| | MUC1-High | MUC1-Low | Wilcox-test |
|---|---|---|---|
| aDC | 0.1498 | 0.1010 | 1.26E-04 |
| Adipocytes | 0.0299 | 0.0570 | 6.73E-10 |
| Astrocytes | 0.0826 | 0.0766 | 8.96E-01 |
| B-cells | 0.0446 | 0.0055 | 7.42E-11 |
| Basophils | 0.0813 | 0.1303 | 8.02E-14 |
| CD4+ memory T-cells | 0.0074 | 0.0004 | 8.98E-14 |
| CD4+ naive T-cells | 0.0119 | 0.0029 | 7.36E-01 |
| CD4+ T-cells | 0.0018 | 0.0001 | 5.18E-02 |
| CD4+ Tcm | 0.0050 | 0.0038 | 5.33E-01 |
| CD4+ Tem | 0.0126 | 0.0060 | 2.86E-03 |
| CD8+ naive T-cells | 0.0240 | 0.0094 | 2.82E-32 |
| CD8+ T-cells | 0.0156 | 0.0026 | 2.02E-05 |
| CD8+ Tcm | 0.0162 | 0.0046 | 1.15E-03 |
| CD8+ Tem | 0.0021 | 0.0004 | 6.15E-01 |
| cDC | 0.0373 | 0.0224 | 1.15E-02 |
| Chondrocytes | 0.0355 | 0.0518 | 5.26E-08 |
| Class-switched memory B-cells | 0.0505 | 0.0407 | 2.12E-01 |
| CLP | 0.0712 | 0.0482 | 2.22E-08 |
| CMP | 0.0010 | 0.0028 | 4.34E-06 |
| DC | 0.0070 | 0.0017 | 2.79E-11 |
| Endothelial cells | 0.0517 | 0.0585 | 6.14E-03 |
| Eosinophils | 0.0038 | 0.0026 | 1.69E-01 |
| Epithelial cells | 0.2288 | 0.2779 | 6.93E-21 |
| Erythrocytes | 0.0000 | 0.0000 | 4.72E-01 |
| Fibroblasts | 0.0403 | 0.0592 | 3.12E-06 |
| GMP | 0.0012 | 0.0021 | 2.73E-02 |
| Hepatocytes | 0.0017 | 0.0031 | 7.57E-10 |
| HSC | 0.1621 | 0.2170 | 1.44E-07 |
| iDC | 0.0378 | 0.0215 | 3.63E-01 |
| Keratinocytes | 0.0280 | 0.0209 | 5.62E-01 |
| ly Endothelial cells | 0.0174 | 0.0211 | 2.47E-04 |
| Macrophages | 0.0365 | 0.0237 | 2.65E-04 |
| Macrophages M1 | 0.0193 | 0.0082 | 1.36E-08 |
| Macrophages M2 | 0.0381 | 0.0390 | 7.23E-02 |
| Mast cells | 0.0072 | 0.0078 | 1.63E-01 |
| Megakaryocytes | 0.0065 | 0.0052 | 2.17E-02 |
| Melanocytes | 0.0092 | 0.0112 | 1.10E-04 |
| Memory B-cells | 0.0135 | 0.0004 | 3.06E-03 |
| MEP | 0.0595 | 0.0322 | 1.46E-26 |
| Mesangial cells | 0.0086 | 0.0122 | 2.43E-03 |

| | | | |
|---|---|---|---|
| **Monocytes** | 0.0075 | 0.0014 | 1.73E-05 |
| **MPP** | 0.0002 | 0.0000 | 1.04E-02 |
| **MSC** | 0.1498 | 0.2129 | 2.19E-10 |
| **mv Endothelial cells** | 0.0238 | 0.0207 | 3.38E-01 |
| **Myocytes** | 0.0042 | 0.0034 | 3.76E-01 |
| **naive B-cells** | 0.0023 | 0.0000 | 1.27E-01 |
| **Neurons** | 0.0061 | 0.0030 | 6.07E-02 |
| **Neutrophils** | 0.0002 | 0.0003 | 3.55E-01 |
| **NK cells** | 0.0000 | 0.0001 | 9.55E-01 |
| **NKT** | 0.0509 | 0.0524 | 2.16E-01 |
| **Osteoblast** | 0.0516 | 0.0300 | 6.43E-09 |
| **pDC** | 0.0110 | 0.0026 | 4.82E-09 |
| **Pericytes** | 0.0619 | 0.0646 | 1.41E-01 |
| **Plasma cells** | 0.0113 | 0.0064 | 1.30E-07 |
| **Platelets** | 0.0002 | 0.0000 | 6.80E-03 |
| **Preadipocytes** | 0.0169 | 0.0162 | 2.15E-01 |
| **pro B-cells** | 0.0222 | 0.0030 | 2.35E-24 |
| **Sebocytes** | 0.0140 | 0.0105 | 2.97E-01 |
| **Skeletal muscle** | 0.0025 | 0.0010 | 3.70E-02 |
| **Smooth muscle** | 0.1645 | 0.1637 | 9.80E-01 |
| **Tgd cells** | 0.0025 | 0.0006 | 1.37E-02 |
| **Th1 cells** | 0.0854 | 0.0464 | 9.44E-16 |
| **Th2 cells** | 0.1900 | 0.0544 | 8.44E-39 |
| **Tregs** | 0.0042 | 0.0034 | 7.32E-01 |
| **ImmuneScore** | 0.0828 | 0.0304 | 2.37E-10 |
| **StromaScore** | 0.0609 | 0.0873 | 6.87E-07 |
| **MicroenvironmentScore** | 0.1437 | 0.1178 | 9.28E-01 |



Yamashita N, et al. J Immunother Cancer 2021; 9:e002115. doi: 10.1136/jitc-2020-002115

**Supplemental Table S3. Analysis of MUC1 association with cell populations in the METABRIC cohort.**

| | MUC1-High | MUC1-Low | Wilcox-test |
|---|---|---|---|
| aDC | 0.2014 | 0.1018 | 3.91E-38 |
| Adipocytes | 0.0101 | 0.0098 | 8.16E-05 |
| Astrocytes | 0.0487 | 0.0387 | 6.37E-03 |
| B-cells | 0.0835 | 0.0288 | 8.96E-22 |
| Basophils | 0.1094 | 0.0781 | 2.36E-11 |
| CD4+ memory T-cells | 0.0336 | 0.0090 | 4.31E-43 |
| CD4+ naive T-cells | 0.0200 | 0.0123 | 8.55E-03 |
| CD4+ T-cells | 0.0038 | 0.0005 | 3.54E-05 |
| CD4+ Tcm | 0.0032 | 0.0036 | 8.82E-01 |
| CD4+ Tem | 0.0077 | 0.0047 | 5.22E-03 |
| CD8+ naive T-cells | 0.0043 | 0.0018 | 3.38E-14 |
| CD8+ T-cells | 0.0449 | 0.0183 | 9.55E-19 |
| CD8+ Tcm | 0.0907 | 0.0512 | 5.99E-20 |
| CD8+ Tem | 0.0152 | 0.0030 | 4.53E-19 |
| cDC | 0.1186 | 0.0772 | 5.64E-06 |
| Chondrocytes | 0.0327 | 0.0370 | 1.12E-04 |
| Class-switched memory B-cells | 0.0316 | 0.0146 | 3.04E-14 |
| CLP | 0.0590 | 0.0419 | 5.28E-29 |
| CMP | 0.0052 | 0.0064 | 1.79E-07 |
| DC | 0.0196 | 0.0062 | 2.63E-31 |
| Endothelial cells | 0.0149 | 0.0132 | 9.61E-01 |
| Eosinophils | 0.0003 | 0.0005 | 1.77E-01 |
| Epithelial cells | 0.1362 | 0.1665 | 7.88E-31 |
| Erythrocytes | 0.0001 | 0.0000 | 7.88E-02 |
| Fibroblasts | 0.0164 | 0.0221 | 4.20E-08 |
| GMP | 0.0062 | 0.0042 | 7.80E-01 |
| Hepatocytes | 0.0011 | 0.0013 | 3.96E-03 |
| HSC | 0.1562 | 0.1586 | 1.74E-01 |
| iDC | 0.1002 | 0.0466 | 1.55E-06 |
| Keratinocytes | 0.0178 | 0.0115 | 2.19E-02 |
| ly Endothelial cells | 0.0090 | 0.0089 | 2.88E-02 |
| Macrophages | 0.0354 | 0.0205 | 1.50E-15 |
| Macrophages M1 | 0.0446 | 0.0222 | 2.46E-36 |
| Macrophages M2 | 0.0078 | 0.0057 | 6.80E-02 |
| Mast cells | 0.0162 | 0.0194 | 1.53E-11 |
| Megakaryocytes | 0.0006 | 0.0006 | 3.78E-01 |
| Melanocytes | 0.0041 | 0.0022 | 1.75E-04 |



Yamashita N, *et al. J Immunother Cancer* 2021; 9:e002115. doi: 10.1136/jitc-2020-002115

| | | | |
|---|---|---|---|
| **Memory B-cells** | 0.0326 | 0.0116 | 2.17E-18 |
| **MEP** | 0.0444 | 0.0326 | 1.14E-23 |
| **Mesangial cells** | 0.0558 | 0.0578 | 1.93E-01 |
| **Monocytes** | 0.0204 | 0.0069 | 3.83E-20 |
| **MPP** | 0.0023 | 0.0015 | 4.23E-01 |
| **MSC** | 0.0215 | 0.0301 | 2.02E-03 |
| **mv Endothelial cells** | 0.0107 | 0.0085 | 4.03E-01 |
| **Myocytes** | 0.0042 | 0.0041 | 3.83E-01 |
| **naive B-cells** | 0.0060 | 0.0019 | 3.24E-13 |
| **Neurons** | 0.0047 | 0.0026 | 5.32E-01 |
| **Neutrophils** | 0.0003 | 0.0004 | 4.22E-01 |
| **NK cells** | 0.0002 | 0.0000 | 1.39E-01 |
| **NKT** | 0.0501 | 0.0690 | 3.54E-19 |
| **Osteoblast** | 0.0092 | 0.0071 | 5.59E-01 |
| **pDC** | 0.0657 | 0.0221 | 1.34E-30 |
| **Pericytes** | 0.0180 | 0.0223 | 2.73E-03 |
| **Plasma cells** | 0.0216 | 0.0105 | 3.85E-17 |
| **Platelets** | 0.0003 | 0.0001 | 8.56E-02 |
| **Preadipocytes** | 0.0319 | 0.0252 | 1.12E-03 |
| **pro B-cells** | 0.0117 | 0.0036 | 4.27E-26 |
| **Sebocytes** | 0.0141 | 0.0109 | 7.88E-01 |
| **Skeletal muscle** | 0.0012 | 0.0007 | 4.65E-03 |
| **Smooth muscle** | 0.3307 | 0.3252 | 9.98E-01 |
| **Tgd cells** | 0.0000 | 0.0000 | 1.81E-02 |
| **Th1 cells** | 0.0324 | 0.0180 | 1.10E-07 |
| **Th2 cells** | 0.2313 | 0.0994 | 3.69E-65 |
| **Tregs** | 0.0034 | 0.0013 | 1.74E-03 |
| **ImmuneScore** | 0.1498 | 0.0677 | 1.81E-27 |
| **StromaScore** | 0.0207 | 0.0225 | 1.97E-05 |
| **MicroenvironmentScore** | 0.1705 | 0.0902 | 9.23E-20 |
